# Supplementary material for: Hybrid Models and Biological Model Reduction with PyDSTool
Source: PLoS Comput Biol. 2012 Aug 9;8(8):e1002628. doi: 10.1371/journal.pcbi.1002628 (PMC3415397; doi:10.1371/journal.pcbi.1002628)
Supplement: Text S4 — Complete source code for the PyDSTool package (version 0.88.120504). Includes API documentation and help files linking to web pages. This file is identical to the current public release on Sourceforge.net. (ZIP) [file pcbi.1002628.s004.zip › PyDSTool/html/PyDSTool.Toolbox.optimizers.line_search-pysrc.html]

xml version="1.0" encoding="ascii"?


PyDSTool.Toolbox.optimizers.line\_search


| Home | Trees | Indices | Help | PyDSTool |
| --- | --- | --- | --- | --- |

Package PyDSTool ::
Package Toolbox ::
Package optimizers ::
Package line\_search

[hide private]

[frames] | no frames]

# Source Code for Package PyDSTool.Toolbox.optimizers.line\_search

```
 1   
 2  # Matthieu Brucher 
 3  # Last Change : 2007-12-12 09:29 
 4   
 5  """ 
 6  Module containing the line searchers 
 7   
 8  Line Searches : 
 9    - SimpleLineSearch 
10      - takes a simple step 
11    - HyperbolicLineSearch 
12      - takes a step of length 1/(1+iterations) 
13    - DampedLineSearch 
14      - searches for a candidate by dividing the step by 2 each time 
15    - BacktrackingSearch 
16      - finds a candidate according to the Armijo rule 
17   
18    - WolfePowellRule 
19      - finds a candidate according to the standard Wolfe-Powell rules 
20    - StrongWolfePowellRule 
21      - finds a candidate according to the strong Wolfe-Powell rules 
22    - GoldsteinRule 
23      - finds a candidate according to the Goldstein rules 
24   
25    - GoldenSectionSearch 
26      - uses the golden section method for exact line search 
27    - FibonacciSectionSearch 
28      - uses the Fibonacci section method for exact line search 
29    - QuadraticInterpolationSearch 
30      - uses the quadratic interpolation method with computation of the gradient at the origin for exact line search 
31   
32    - AdaptiveLastStepModifier 
33      - modifies the last step length depending on the last direction and gradient and current gradient and direction 
34    - FixedLastStepModifier 
35      - modified the last step length with a fixed factor 
36  """ 
37   
38  from simple_line_search import * 
39  from hyperbolic_line_search import * 
40  from damped_line_search import * 
41  from backtracking_search import * 
42   
43  from wolfe_powell_rule import * 
44  from strong_wolfe_powell_rule import * 
45  from goldstein_rule import * 
46   
47  from golden_section import * 
48  from fibonacci_section import * 
49  from quadratic_interpolation import * 
50  from cubic_interpolation import * 
51   
52  from adaptive_last_step_modifier import * 
53  from fixed_last_step_modifier import * 
54   
55  from scaled_line_search import * 
56   
57  line_search__all__ = ['SimpleLineSearch', 'HyperbolicLineSearch', 'DampedLineSearch', 'BacktrackingSearch', 
58                        'WolfePowellRule', 'StrongWolfePowellRule', 'GoldsteinRule', 
59                        'GoldenSectionSearch', 'FibonacciSectionSearch', 'QuadraticInterpolationSearch', 'CubicInterpolationSearch', 
60                        'AdaptiveLastStepModifier', 'FixedLastStepModifier', 'ScaledLineSearch'] 
61  __all__ = line_search__all__ 
62
```

  


| Home | Trees | Indices | Help | PyDSTool |
| --- | --- | --- | --- | --- |

|  |  |
| --- | --- |
| Generated by Epydoc 3.0.1 on Fri May 4 15:24:14 2012 | http://epydoc.sourceforge.net |
